# Surgical management of leprosy-associated neuropathy in Pakistan: Restoring function and reducing disability in the post-elimination era: A narrative review

**DOI:** 10.12669/pjms.42.(11AASC).15800

**Published:** 2026-04

**Authors:** Faheela Tamseel, Sahla Waqas, Inam Ul Haq

**Affiliations:** 1 Faheela Tamseel, MBBS, Liaquat National Hospital and Medical College, Karachi, Pakistan; 2 Sahla Waqas, MBBS, King Edward Medical University, Lahore, Pakistan; 3 Inam Ul Haq, MBBS, Rashid Latif Medical College, Lahore, Pakistan

**Keywords:** Leprosy, Neuropathy, Post-elimination era, Post-surgical rehabilitation, Surgery

## Abstract

**Objectives:**

To narratively review the role of surgical interventions in the management of leprosy-associated neuropathy in Pakistan, focusing clinical indications, timing, and outcomes of nerve decompression, nerve abscess management, and reconstructive procedures. This review also aimed to highlight gaps in local evidence, compare Pakistani practices with global standards, and assess how surgical care can be integrated into national leprosy control efforts to reduce disability in the post-elimination era.

**Methodology:**

Literature search was conducted (PubMed, Embase, Cochrane; 1980–2025) to provide a broad overview of the epidemiology, medical management, rehabilitation, and surgical treatment of leprosy-associated neuropathy. Studies describing surgical indications, techniques, outcomes, or rehabilitation were emphasized. Publications focused exclusively on medical therapy without neurological outcomes were excluded. Pakistani studies were prioritized, while international literature was included to contextualize local practice and supplement limited national data.

**Results:**

Surgical services in Pakistan are largely limited to specialized centres such as MALC and AKUH. Nerve decompression, tendon transfers, and reconstructive procedures have the potential to restore function and prevent ulcers, with functional recovery rates in comparable low-resource settings. Early surgical intervention combined with rehabilitation yields the best outcomes in most of the articles. International models demonstrate that integration of surgical care into national programs is feasible and effective, even in resource-constrained environments.

**Conclusion:**

The reviewed evidence indicates that surgical intervention with rehabilitation offers optimal outcomes for leprosy-associated neuropathy. In Pakistan’s post-elimination era, prioritizing disability prevention through expansion of surgical services, strengthening reconstructive training, and integrating rehabilitation into national leprosy programs is essential to reducing long-term disability and stigma.

## INTRODUCTION

Leprosy, also known as Hansen’s disease, is a chronic infectious disease caused by *M.leprae*, primarily affecting the skin, peripheral nerves, and mucosa of the upper respiratory tract.^1^ The organism’s predilection for Schwann cells leads to inflammation and fibrosis of peripheral nerves, resulting in sensory loss, motor weakness, and deformities that contribute to long-term disability.^2^ Although a multidrug therapy (MDT) effectively cures the infection, nerve damage often continues during or after treatment, making leprosy-associated neuropathy the leading cause of functional impairment and disability. Globally, around 133,000 new cases of leprosy were reported in 2023, with over 10% presenting with Grade-2 disability, reflecting ongoing transmission and delayed diagnosis.^3^

Pakistan achieved the World Health Organization (WHO) elimination threshold in 1996, defined as fewer than one case per 10,000 population, but elimination did not equate to eradication.^4^ A recent qualitative study highlights that national data from Pakistan indicate approximately 250–300 new cases are detected annually.^5^ Another study shows that between 2001 and 2023, new adult cases dropped by 75%, from 878 cases to 220 cases annually, and child cases by 83% dropping from 93 to 16 cases.^6^ The rise in the figures despite elimination indicates persistent gaps in early detection, nerve function monitoring, and disability prevention.

Among the disabilities caused by leprosy, neuropathy remains the single largest contributor to lifelong disability and social stigma in leprosy, limiting independence and employability.^7^ Surgical interventions such as nerve decompression, tendon transfer, and reconstructive correction of deformities have shown promising results in restoring movement, preventing contractures, and significantly improving functional and psychosocial outcomes.^8^

Despite the historical success of Pakistan’s leprosy elimination, the long-term functional burden of neuropathy and deformity remains under-addressed. While international literature recognizes surgery as an integral component of leprosy care, there is currently no comprehensive synthesis of existing data on surgical approaches, outcomes, and health system barriers in Pakistan. In the post-elimination era, where disease incidence is low but disability persists, synthesizing this review is crucial to guide service planning, training, and integration of surgical rehabilitation into primary leprosy care. This narrative review, therefore, aimed to summarize recent data on the surgical management of leprosy-associated neuropathy in Pakistan, identify gaps in practice and policy, and propose strategies to strengthen restorative and disability-reduction services.

## METHODOLOGY

A systematic search of PubMed/MEDLINE, Embase, and the Cochrane Library was conducted for studies using the Boolean string: leprosy (“leprosy,” “Hansen’s disease,” “leprosy neuropathy”), surgical interventions (“surgery,” “surgical management,” “reconstructive surgery,” “nerve decompression,” “tendon transfer,” “neurolysis”), and context (“Pakistan,” “South Asia”). Due to the limited availability of recent peer-reviewed studies on surgical management of leprosy-associated neuropathy, especially from low-resource settings like Pakistan, the review also incorporates older, foundational studies where necessary to describe surgical techniques, outcomes, and functional results, including studies from 1980-2025. This approach allows for a comprehensive synthesis of evidence while acknowledging the scarcity of contemporary regional data.

Eligible studies included human research addressing the surgical management or rehabilitation of leprosy-associated neuropathy, including randomized trials, observational studies, case series, reviews, and expert consensus. Excluded were studies of animal-only research, conference abstracts without full data, and publications lacking clinically applicable information.

The review was structured and reported in alignment with the Scale for the Assessment of Narrative Review Articles (SANRA), ensuring transparency of literature search, and appropriate referencing. Emphasis was placed on evidence with implications for disability prevention, highlighting surgical strategies that can be integrated into clinical care in Pakistan’s post-elimination context.

### Current status of leprosy management in Pakistan:

### Epidemiologic Overview:

Although Pakistan achieved WHO’s elimination target in 1996, leprosy persists in certain regions, particularly Sindh, Balochistan, and Khyber Pakhtunkhwa.^6^ Rural and underserved populations are disproportionately affected, with adult males comprising the majority of new cases and children under 15 representing a smaller but persistent fraction.^9^

Newly diagnosed patients often present with advanced neuropathy and visible deformities, with about one-third exhibiting Grade-2 disability at diagnosis, reflecting delayed detection.^9^ The Marie Adelaide Leprosy Centre (MALC) and its satellite clinics remain the main centers for diagnosis, treatment, and rehabilitation, providing MDT, physiotherapy, and surgical referral.^6^,^9^ Longitudinal data indicate a gradual decline in overall case numbers, but the proportion of patients presenting with disability remains high, underscoring the ongoing need for integrated rehabilitation and early referral programs.

### Current Treatment and Recovery:

Multi drug therapy (MDT) in Pakistan follows the internationally recommended module of WHO, which includes the combination of rifampicin, dapsone, and clofazimine.^1^ The cure of *M.leprae* infection is >95% with this regimen demonstrating high bacteriological cure rates, and effective elimination of *M.Leprae* infection.^7^

Despite microbiological cure, functional recovery from neuropathy remains limited, mainly due to delayed diagnosis and established nerve damage at presentation. Corticosteroids are given for acute neuritis to reduce inflammation and prevent further nerve injury; physiotherapy and basic rehabilitation services are also made available at some centers to maintain joint mobility and muscle strength.^9^

Although the relapse rate was low, the proportion of patients having Grade-2 disability remained considerable (~20–25%), indicating persisting challenges in the prevention of disability and restoration of function. These results further emphasize how necessary the combination of surgical and rehabilitative approaches is to complement MDT for the optimization of long-term outcomes.^7^,^9^

### Use of Surgical Treatment in Pakistan:

Surgical rehabilitation services for leprosy-associated neuropathy are generally limited to specialist centres in Karachi, Pakistan, including Marie Adelaide Leprosy Centre (MALC) and Aga Khan University Hospital (AKUH). MALC’s centre and its satellite clinics continue to play a vital role in diagnosis, treatment and rehabilitation pathways; however, the number of reconstructive surgeries, including nerve decompression, tendon transfers and ulcer reconstruction, remains limited and is often implemented through non-governmental programmes rather than a national surgical referral pathway.^6^,^10^

Despite the presence of evidence from international literature showing functional improvement post-surgery, there is no structured surgical referral system in Pakistan within the national leprosy control programme; there also exists a significant training gap where general and plastic surgeons rarely receive any specific exposure to leprosy-specific reconstructive techniques.^10^,^11^ The follow-up and rehabilitation pot-surgery are irregular, thereby reducing the long-term functional gains and participation outcomes for most patients.^12^

**Fig.1:**
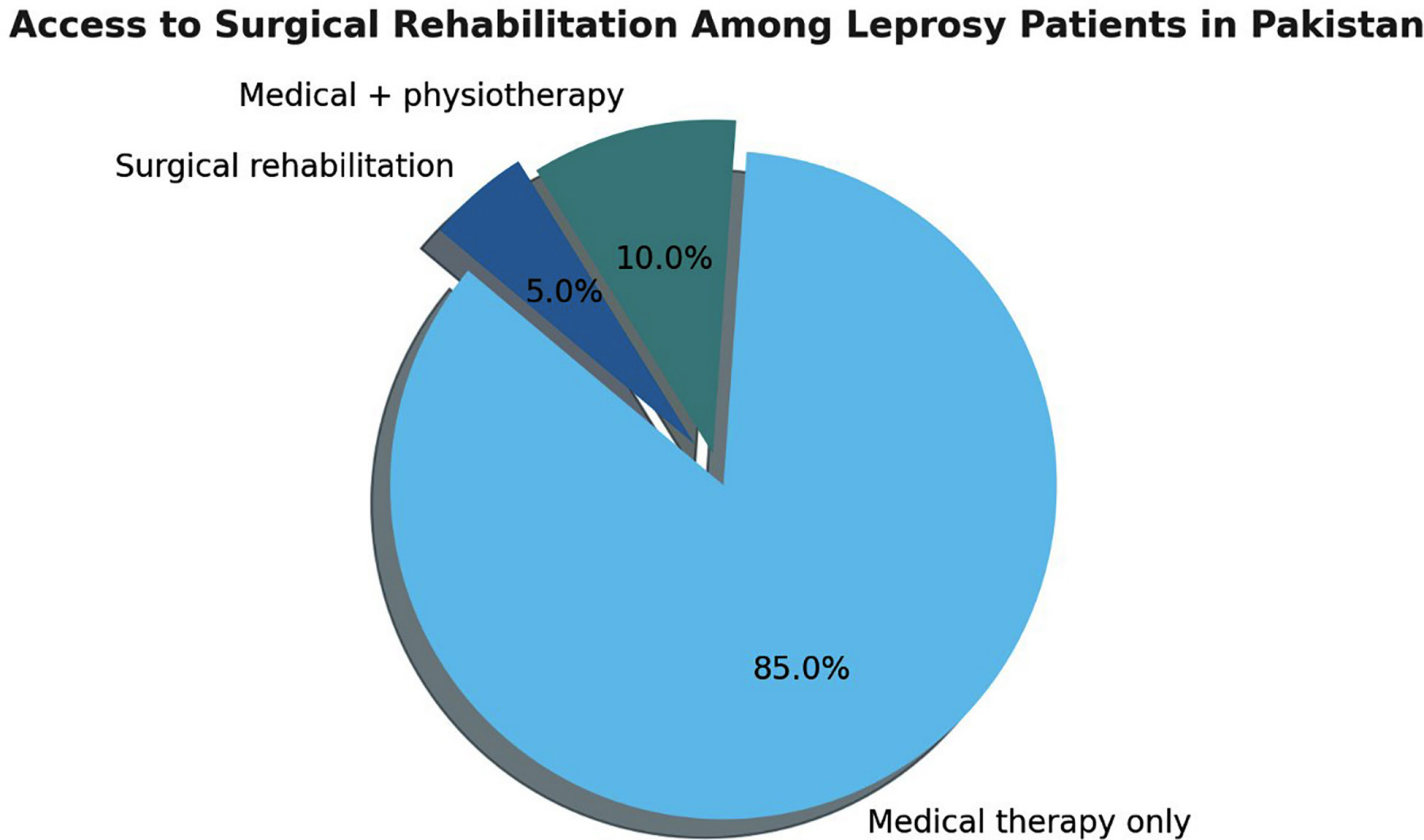
Access to surgical rehabilitation among treated leprosy patients in Pakistan (approximate distribution based on WHO Global Leprosy Update 2024 and MALC Annual Report 2024).

Fig.1 demonstrates approximate percentage according to WHO and MALC 2024 reports that most of the leprosy patients receive only medical treatment while surgical interventions remain limited to specific centres only.

**Fig.2:**
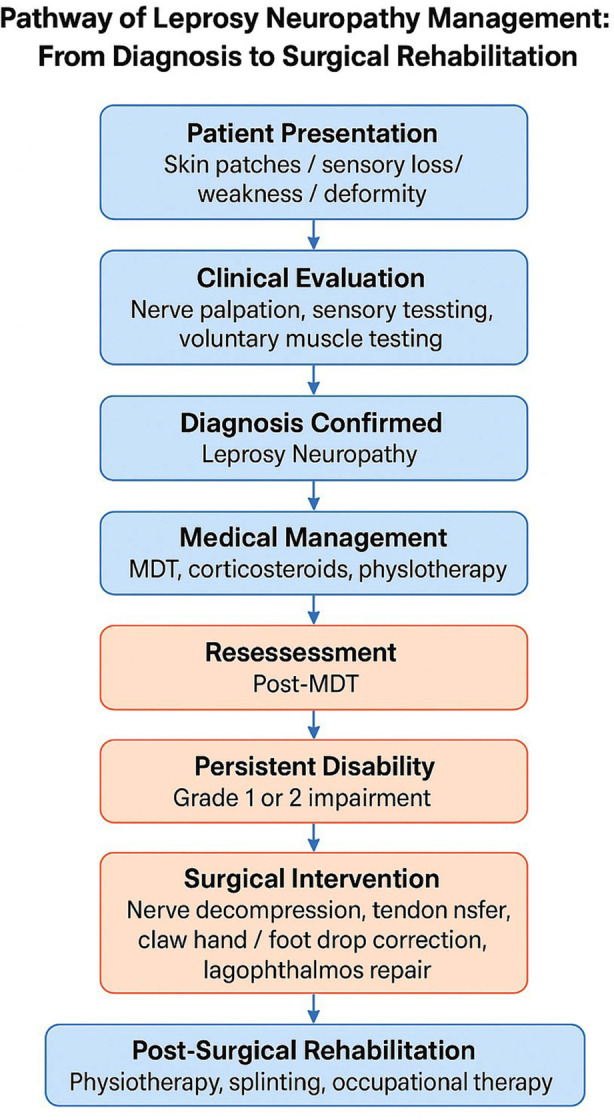
Pathway of Leprosy Neuropathy Management: From Diagnosis to Surgical Rehabilitation.

### Global perspective on surgical management of leprosy neuropathy:

Globally, several countries have successfully integrated reconstructive and nerve-sparing surgeries into their leprosy programs, demonstrating strong functional outcomes and improved quality of life.

In India, where the disease burden is still highest, reconstructive leprosy surgery is routinely practiced in specialized centres such as the Christian Medical College (Vellore) and Karigiri Leprosy Hospital, the Netherlands Leprosy Relief network. Nerve decompression, tendon transfer, and flap surgeries are some of the procedures performed. Published data report functional improvement rates of 70–90%, especially when surgery is performed early, before irreversible fibrosis. India’s integrated model combining surgery, physiotherapy, and community-based rehabilitation is recognized by WHO as a model of post-elimination care.^13^

Reconstructive surgery in Brazil is a part of the Unified Health System; hospitals such as Instituto Lauro de Souza Lima and Fiocruz Foundation are providing multidisciplinary care. Surgical outcomes have been favourable, with high patient satisfaction, pain relief, and restored function.^14^ Another successful model involves Anandaban Hospital and International Nepal Fellowship, known as INF, which offers low-cost reconstructive operations using mobile surgical camps and demonstrate that community physiotherapy and follow-up programmes can sustainably improve hand strength, gait, and prevent recurrent ulcers.^15^ By comparison, sub-Saharan African countries like Ethiopia, Nigeria, and Tanzania depend highly on short-term reconstructive surgery missions, greatly improving mobility and reduce stigma.^16^

**Table-I:** Global comparison of surgical interventions and outcomes.

Country / Region	Common Surgical Procedures	Reported Outcomes	Notable Centres / Programs
India^14^	Nerve decompression, tendon transfer, claw hand correction, lagophthalmos repair	70–90% functional recovery; improved hand and foot mobility; decreased ulcer recurrence	CMC Vellore, Karigiri Leprosy Mission Hospitals
Brazil^15^	Nerve decompression, reconstructive plastic surgery, joint stabilization	>80% improvement in sensory and motor function; strong rehab integration	Instituto Lauro de Souza Lima, Fiocruz
Nepal^16^	Low-cost mobile surgical camps; tendon balancing; reconstructive surgery	Good long-term hand and foot outcomes; community reintegration success	Anandaban Hospital (INF), The Leprosy Mission Nepal
Africa (Ethiopia, Nigeria)^17^	Limited reconstructive efforts; occasional mission-based tendon transfer	Functional improvement noted, but lack of follow-up limits durability	ALERT Hospital, Addis Ababa
Pakistan	Minimal reported data; occasional tendon release and ulcer reconstruction at MALC		Marie Adelaide Leprosy Centre (MALC), Karachi

**Table-II:** Major barriers hindering the uptake of surgical rehabilitation for leprosy neuropathy in Pakistan and evidence-based strategies to overcome them.

Barrier Category	Specific Barriers	Potential Interventions / Solutions
Health-system limitations	Absence of surgical units within leprosy control programs; poor referral pathways	Integrate reconstructive surgery into National Leprosy Control Programme; create regional referral hubs in Sindh and KP
Human-resource gap	Lack of trained reconstructive and peripheral-nerve surgeons	Initiate short-term fellowship programs with African or Nepali centers; include nerve decompression training in surgical curricula
Financial constraints	Limited government and NGO funding for post-MDT disability care	Establish public–private partnerships; allocate dedicated rehabilitation budget lines
Awareness & stigma	Patients unaware that surgical options exist; social stigma discourages care-seeking	Nationwide awareness through media, community health workers, and rehabilitated patient ambassadors
Post-operative follow-up	Weak rehabilitation and physiotherapy linkage	Introduce community-based rehab follow-up programs; tele-rehab for rural patients
Data & monitoring	No national registry or surgical outcome tracking	Develop a digital leprosy disability and surgery registry; periodic outcome audits

Through all these experiences, evidence is consistent that surgical correction when combined with postoperative rehabilitation and social reintegration significantly reduces disability and stigma. The success from these countries demonstrate that this is possible even in low-resource settings if surgical services are formally integrated into national leprosy programs, underpinning the urgent need to establish a national reconstructive and rehabilitative framework in Pakistan.

### Indications for surgical intervention:

Most leprosy-related nerve damage is initially managed medically, typically with corticosteroids, anti-inflammatory therapy, and physiotherapy. However, surgery becomes essential when neurological deficits persist, worsen, or result in functional impairment despite adequate medical treatment. The primary objective of sugery is to prevent or minimize disability, alleviate pain, and restore function.

### The key indications of surgery include:

### Progressive or unresponsive nerve compression:

Persistent inflammation or fibrosis of major peripheral nerves causing sensory loss or motor weakness. Despite taking steroids for at least four weeks, there is ongoing nerve discomfort and sensory/motor loss; decompression or neurolysis may be necessary.^17^

### Entrapment neuropathy:

Thickened nerves that produce compression symptoms, such as carpal/tarsal tunnel syndrome; decompression is suggested.^17^

### Tibial neuritis:

Involvement of the lower limb nerves resulting in discomfort or loss of motor function.^18^

### Established deformity or functional loss:

Conditions such as claw hand, foot drop, or lagophthalmos that impair function or lead to secondary complications. Non-healing ulcers, recurrent infections, or joint instability that do not respond to conservative care, risking further tissue loss, thus requires reconstructive surgery or tendon transfer.^19^

### Chronic neuropathic pain:

Decompression or neurolysis may be helpful for pain that is not alleviated by medication.^20^

Timely referral for surgery is crucial, as early intervention before irreversible fibrosis or deformity leads to better functional outcomes, preserves independence, and reduces long-term disability and stigma.

### Surgical approaches and techniques:

Surgery is a key component of managing long-standing leprosy neuropathy, particularly when medical therapy cannot reverse nerve damage or correct deformities. Procedures such as nerve decompression, tendon transfers, and reconstructive surgeries help restore function, prevent ulcers, and improve quality of life. This section outlines the main techniques used globally and their relevance and outcomes in low-resource settings.

### Nerve Decompression:

Nerve decompression is recommended when pharmacological treatment, such as corticosteroids, is insufficient to alleviate leprosy patients’ chronic nerve pain and inflammation, leading to a persistent neuritis or nerve abscess.^21^ Decompression improves overall limb function and lessens disability in leprosy-associated neuropathy. The most often decompressed nerves are the ulnar, median, posterior tibial, peroneal, and facial.^22^

Observational series from Brazil show that over 80 % of operated nerves were maintained or improved in function after decompression.^15^ A 2021 Indian study of median-nerve decompression in leprosy found better outcomes when surgery plus steroids compared to steroids alone.^18^ While a Cochrane review concluded that RCT evidence is very low quality, the non-randomised data support benefit in experienced hands.^23^

### Tendon transfer procedure:

After leprosy causes irreversible paralysis, tendon transfer treatments are crucial for improving hand and foot function by addressing significant abnormalities. Tendon transfers are predicated on the idea that functional components are reorganised into the most advantageous working configuration rather than creating anything new. This is accomplished by replacing a damaged or non-functioning muscle-tendon unit with a tendon from a working muscle. We commonly use the tendon transfer procedure for the claw hand, foot drop and lagopthalomos.

The Stiles-Bunnel flexor digitorum superficialis (FDS) tendon transfer or lumbrical-interosseous transfer are often utilised techniques in cases of claw hand improve pinch/grip and restore MCP flexion with IP extension by rerouting an extrinsic flexor tendon to function as an intrinsic extensor mechanism at the MCP joints.^24^,^25^

The common surgical treatment for foot drop is posterior tibial tendon transfer (PTT), by rerouting the posterior tibial tendon to the dorsum of the foot, the transfer restores active dorsiflexion and enables patients to resume a more natural heel-to-toe stride.^26^

Temporalis transfer and gold weight implants with tarsal strip and eyelid surgery provide reliable eyelid closure, preventing corneal complications and improving quality of life.^27^

In one Nepalese series of 31 patients with triple-nerve paralysis, excellent/good results were obtained in 93% for wrist extension, 85% for finger extension and 63% for thumb opposition after tendon transfers.^28^

As all the processes prevent permanent deformities, simplify motor retraining, and reduces the long-term need for orthoses, early surgical intervention combined with postoperative physiotherapy is crucial for achieving better functional outcomes.

### Reconstructive and Corrective Surgeries:

Reconstructive and corrective surgeries are vital for managing chronic ulcers, contractures, and tissue loss in leprosy, aiming to restore function, prevent recurrence, and reduce social stigma. Many surgeons worldwide have reported their techniques with advancements in the use of grafts and skin flaps with success on closure of the lesions due to the high rate of recurrence of these lesions by conservative methods. Surgical techniques include a variety of ways like: Skin grafts; surgical debridement; use of Bipediculated local flaps; posterior tibial nerve neurolysis; plantar flap transposition with the neural plexus and blood supply; autologous follicular and smashed dermal graft.^29^

Series report that reconstructive surgery in neuropathic ulcer patients reduces recurrence and improves functional mobility. For example, a sensory nerve transfer series in India showed complete ulcer healing in all feet at 6 months follow-up.^30^ These interventions not only promote healing and reduce recurrence but also play a crucial role in restoring dignity and reducing the social stigma associated with leprosy-related disabilities.

### Post operative rehabilitation and outcomes:

Effective leprosy rehabilitation depends on a multidisciplinary effort where surgery works hand-in-hand with physiotherapy, occupational therapy, podiatry, and counseling. The surgeon functions as part of a coordinated team with the patient at its center, following the WHO’s multidisciplinary care model.^24^

Although reconstructive surgery is cost-effective and life-changing in terms of restoring essential function and dignity for leprosy patients, physiotherapy is equally vital in treating nerve inflammation, correcting paralytic contractures, healing ulcers, and preserving movement after surgery.^31^

Rehabilitation in leprosy follows a structured progression comprising three stages^32^:

### Immobilization phase (2–3 weeks):

Initially, the limb is immobilized with splints and casts to protect tendon transfers or decompressed nerves and allow wound healing.

### Mobilization phase:

As recovery progresses, active and passive exercises are performed to mobilize the joints and prevent contractures and stiffness.

### Strengthening phase:

Finally, muscle strength is restored with the help of resisted exercises to retrain transferred tendons and improve functional control.

This stepwise approach ensures maximum healing and a return to independence. The use of protective footwear is essential for leprosy patients because they can develop plantar ulcers due to sensory loss and repeated pressure. A newly developed computer-aided orthotic device can help redistribute plantar pressure, thereby reducing the risk of recurrent ulcers and deformities. It is lightweight, comfortable, cosmetically acceptable and significantly improves patient compliance.^33^

Reported outcomes from India and specialized centers (e.g., MALC) show that 70–90% of patients regain meaningful functional independence after tendon transfers or decompression when rehabilitation is consistent and supervised. However, long-term follow-up remains a major challenge due to socioeconomic barriers, limited accessibility to physiotherapy, and poor continuity of care in endemic regions.^34^

To address these gaps, community-based rehabilitation and peer-support models have proven effective in improving adherence, early detection of complications, and overall quality of life, especially relevant in low-resource settings where formal rehabilitation services are limited

### Challenges and barriers in Pakistan:

Pakistan has achieved remarkable progress in its journey toward zero leprosy, its complete elimination is hindered by many barriers. Since 2001, leprosy cases have declined by over 75%; however, about 20% of new patients still present with visible or grade two disabilities, pointing towards a delay in diagnosis. This is likely due to weak referral systems and gaps in leprosy care, especially in disease hotspots like Karachi. The lack of a unified national leprosy program forces NGOs and provincial units to carry most of the care and control burden. Poor surveillance, limited resources, a shortage of experts, and ongoing stigma toward leprosy continue to hinder control efforts.^6^

Patient data is recorded on paper and not digitally, and thus cannot be tracked or used properly, especially the data from remote areas. Moreover, prophylactic treatment is often not provided to the close contacts of leprosy patients, leading to the development of new cases.^35^ Primary health care providers often fail to diagnose it correctly due to a lack of training. Impoverished people from remote areas often remain untreated because they are unable to access or afford necessary care. Moreover, the lack of research on the latest detection and treatment methods further limits progress in disease control.^5^

Moreover, Pakistan’s healthcare system is severely underfunded, receiving just 0.4% of GDP which is far below global standards.^36^ There’s especially a gap in the availability of advanced surgical care for all. Evidence from a local study shows that access to surgical care is hindered by a complex mix of factors, including lack of knowledge, cost constraints, and gender disparities, with women facing significantly longer delays.^37^

Severe shortages of certified surgeons and anaesthetists leave many hospitals unable to perform essential procedures. Many essential medicines, lab tests, and imaging services simply aren’t available when needed. Mismanagement and uneven workforce distribution waste the few resources that do exist. These barriers together leave millions without timely, life-saving surgical care.^38^

### Future directions and recommendations:

To control the burden of leprosy in Pakistan, there is a need to strengthen the leprosy surveillance systems by shifting towards the digitalization of records and introducing Geographic Information System (GIS)–based mapping. This way, by identifying the disease heavy regions, health teams can do targeted efforts by conducting skin camps and home visits in those particular areas. Moreover, there is a need to properly train the healthcare workers and to improve the diagnostic laboratory facilities, in order to timely detect and manage the disease. Integrating leprosy treatment within broader skin NTD programs might enhance leprosy control and ensure adequate supply of care. Limited access to prophylactic immunization and medications further creates a gap in leprosy control. There is a need to ensure the equitable supply of vaccines and medicines to everyone. Efforts should be focused upon preventing lifelong disabilities and managing complications.^6^,^39^

It has already been established that surgical care is an important part of leprosy management, especially to correct deformities and restore function. Therefore, it is important to make sure that good quality surgical care is accessible to patients.

In Pakistan, because of the severely underfunded healthcare system and low salaries, there is a shortage of specialized surgeons and highly skilled healthcare professionals. Moreover, healthcare programs like Sehat Sahulat often do not adequately cover surgical costs, creating further barriers for patients to access timely care^3^. Future attention should be focused towards increasing healthcare budgets, improving salaries to retain skilled workers, and overcoming the shortages in infrastructure and essential medical supplies.

### Strengths:

This narrative review is the first comprehensive review in Pakistan on surgical management of leprosy-associated neuropathy, synthesizing local studies, national program reports, and relevant international evidence. Inclusion of older studies provided insights into foundational surgical techniques and long-term outcomes applicable in resource-limited settings.

### Limitations:

A systematic review or meta-analysis was not feasible due to heterogeneity in study designs, variability in outcome measures, small sample sizes, and limited recent data; therefore, exact percentages or pooled estimates of surgical outcomes could not be calculated. Importantly, no national study on surgical outcomes in leprosy-associated neuropathy currently exists in Pakistan, highlighting a significant knowledge gap. Limitations include reliance on evidence from specialized centers, which may limit generalizability.

## CONCLUSION

Pakistan has eliminated leprosy as a public health issue; nonetheless, disability resulting from neuropathy continues to pose a significant barrier. Surgical intervention via nerve decompression, tendon transfer, and reconstructive techniques can rehabilitate function and restore dignity to impacted persons. However, only a small number of centers have access. It is crucial to enhance training, include surgical rehabilitation into national programs, and guarantee early physiotherapy intervention. In order to truly eradicate leprosy, the disease must be cured together with its infirmities.

### Recommendations:

Prospective, multicenter studies with standardized functional assessments are recommended to better define surgical indications, timing, and outcomes. Integrating surgical care with rehabilitation into national leprosy programs will improve disability prevention, enhance patient quality of life, and provide a clearer understanding of persisting disabilities in the post-elimination era.

### Author`s contribution:

**FT** did literature search, study design, figures, manuscript writing and responsible for integrity of study. **SW** did topic Selection, literature search, manuscript writing, **IUH** did literature Search, manuscript writing. All authors have read and approved the final version of the manuscript.
